# Functional MRI of Auditory Responses in the Zebra Finch Forebrain Reveals a Hierarchical Organisation Based on Signal Strength but Not Selectivity

**DOI:** 10.1371/journal.pone.0003184

**Published:** 2008-09-10

**Authors:** Tiny Boumans, Sharon M. H. Gobes, Colline Poirier, Frederic E. Theunissen, Liesbeth Vandersmissen, Wouter Pintjens, Marleen Verhoye, Johan J. Bolhuis, Annemie Van der Linden

**Affiliations:** 1 Bio-Imaging Lab, University of Antwerp, Antwerp, Belgium; 2 Behavioural Biology and Helmholtz Institute, Utrecht University, Utrecht, The Netherlands; 3 Helen Wills Neuroscience Institute and Psychology Department, University of California, Berkeley, California, United States of America; 4 Vision Lab, University of Antwerp, Antwerp, Belgium; Smithsonian Institution, United States of America

## Abstract

**Background:**

Male songbirds learn their songs from an adult tutor when they are young. A network of brain nuclei known as the ‘song system’ is the likely neural substrate for sensorimotor learning and production of song, but the neural networks involved in processing the auditory feedback signals necessary for song learning and maintenance remain unknown. Determining which regions show preferential responsiveness to the bird's own song (BOS) is of great importance because neurons sensitive to self-generated vocalisations could mediate this auditory feedback process. Neurons in the song nuclei and in a secondary auditory area, the caudal medial mesopallium (CMM), show selective responses to the BOS. The aim of the present study is to investigate the emergence of BOS selectivity within the network of primary auditory sub-regions in the avian pallium.

**Methods and Findings:**

Using blood oxygen level-dependent (BOLD) fMRI, we investigated neural responsiveness to natural and manipulated self-generated vocalisations and compared the selectivity for BOS and conspecific song in different sub-regions of the thalamo-recipient area Field L. Zebra finch males were exposed to conspecific song, BOS and to synthetic variations on BOS that differed in spectro-temporal and/or modulation phase structure. We found significant differences in the strength of BOLD responses between regions L2a, L2b and CMM, but no inter-stimuli differences within regions. In particular, we have shown that the overall signal strength to song and synthetic variations thereof was different within two sub-regions of Field L2: zone L2a was significantly more activated compared to the adjacent sub-region L2b.

**Conclusions:**

Based on our results we suggest that unlike nuclei in the song system, sub-regions in the primary auditory pallium do not show selectivity for the BOS, but appear to show different levels of activity with exposure to any sound according to their place in the auditory processing stream.

## Introduction

For successful vocal communication, our brain needs to process external sounds continuously from the acoustic environment during speaking. It is essential to monitor feedback of one's own voice, in order to detect errors in vocal production that should be corrected to stabilize speech. As a consequence, our brain needs to distinguish between self-generated and externally generated auditory inputs. Songbirds (Passeriformes oscines) share with humans the capacity to produce learned vocalisations [1], [2] that can be used for individual recognition, mate attraction and territorial defense [3]. Juvenile zebra finch (*Taeniopygia guttata*) males learn their song from an adult conspecific male (a tutor) and match their vocalisations to the memory of this song [4], [5]. Partly favoured by the strong dependence on auditory feedback for song learning and maintenance, birdsong is a prominent model system for the study of speech acquisition.

A number of interconnected forebrain nuclei in songbird brain, known collectively as the song system, are involved in sensorimotor learning and song production [6]–[8]. Auditory perception and processing involve brain regions outside the song system, including the primary auditory thalamo-recipient Field L, and higher order auditory areas in the pallium, the caudomedial nidopallium (NCM) and caudal mesopallium (CM) [9]–[19]; for reviews see [20], [21]. Most neurons in the avian song system respond preferentially to playback of the bird's own song (BOS) [22]–[24]. Neurons in the primary auditory pallium, Field L, do not show this degree of specificity and instead show a broad preference for sounds with natural sound statistics [25]–[27]. Between these two processing stages, neurons in the secondary auditory regions NCM and CM exhibit selective responses to conspecific song, and this selectivity is affected by recent and past experience with these sounds [9], [10], [14], [17], [28].

Determining which regions show preferential responding to BOS is important, as neurons sensitive to self-generated vocalisations could mediate auditory feedback that is necessary for song learning and maintenance.

We used blood oxygen level-dependent functional magnetic resonance imaging (BOLD fMRI) to re-examine the hierarchical processing of BOS and conspecific song in the primary auditory pallium of the zebra finch brain. In a previous study, we investigated BOS selectivity over conspecific songs in a tilted coronal slice passing through a relatively anterior region of CM, a central region of Field L (mainly L2a) and a caudal region of NCM. In this previous set of scans, we failed to discover any significant BOS selectivity [29] or hemispheric differences. One goal of the present study was to continue the search for BOS selective regions by scanning in the orthogonal direction, i.e. the sagittal plane. This plane passed through the regions L2a, L3 and NCM, as well as caudal L2b and the caudal region of the medial CM (CMM) that were not investigated in our first study. Because of its anatomical connections, the sub-region L2b may be involved in an intermediary processing step between L2a and the secondary auditory regions, and could thus be the original locus from where BOS selectivity emerges. To test this hypothesis, in the present experiment we measured the global neural activity elicited by BOS and conspecific song in sub-regions L2a and L2b. The second goal of this study was to examine the more general selectivity in characteristic acoustical features found in song over synthetic sounds as it has been observed at the single neuron level [26], [30], [31]. In our previous fMRI study, we performed such analysis by comparing responses to normal conspecific song with those obtained in response to presentation of spectrally or temporally filtered songs. In this paper, we tested an alternative manipulation that had previously been used extensively in single unit studies reviewed in [32]. We compared responses to normal BOS with reversed BOS and BOS where the order of syllables was randomized. We also compared BOS responses with a synthetic song that has similar spectro-temporal modulations but random phase modulations. All these manipulations preserve the overall frequency power spectrum of the signal as well as some second order statistics of the temporal and spectral envelope of the sound but disrupt the characteristic higher spectro-temporal structure found in natural song.

## Materials and Methods

### Subjects

Adult male zebra finches (*Taeniopygia guttata castanotis*, n = 5, 12–20 g body weight) served as subjects for this experiment. The birds were obtained from local suppliers and were kept in the laboratory aviaries with unrestricted access to food and water, temperature between 20°C and 25°C, and natural light/dark rhythm. Experimental procedures were in agreement with the Belgian laws on the protection and welfare of animals and had been approved by the ethical committee of the University of Antwerp (Belgium).

Zebra finches initially received an intramuscular injection in the pectoral muscles of 25 mg/kg ketamine (Ketalar, 50 mg/ml; Parke-Davis, Zaventem, Belgium) and 2 mg/kg medetomidine (Domitor, 1 mg/ml; Orion Pharma, Espoo, Finland). After 30 minutes, medetomidine was continuously infused at a rate of 0.02 ml/h through a catheter positioned in the chest muscle. This allowed the birds to be steadily anesthetized for a minimum of 8 hours. The anesthetized birds were immobilized in a non-magnetic, custom-made head holder composed of a beak mask and a circular radio-frequency (RF) surface antenna (diameter 15 mm) tightly placed around the bird's head above both ears and eyes. Body temperature, respiration rate and amplitude, and expired pCO_2_ were constantly monitored during our experiments. Body temperature was continuously monitored with a cloacal temperature probe (SA-Instruments, Stony Brook, NY) and maintained at 40.3±0.3°C (mean±SD) by a cotton jacket and a water-heated pad connected to an adjustable heating pump (EX-111; Neslab Instruments, Newington, NH). Respiration rate and amplitude were monitored with a small pneumatic sensor (SA-Instruments) positioned under the bird. The expired pCO_2_ was measured by a small tube fixed to the stereotaxic mask and connected to a CO_2_ analyzer (Capstar-100; CWI, Diss Norfolk, UK). The pCO_2_ fluctuations measured during the experiments were almost nonexistent.

### Auditory stimulation

#### Bird's own song recording

The birds were placed individually in soundproof isolation chambers (115×115×205 cm) to record their undirected songs. Recordings were made using a Sennheiser MKH50 P48 microphone (Sennheiser Electronic KG, Wedemark, Germany) and a PC with Avisoft Recorder software (Berlin, Germany). All bird sounds were automatically recorded for approximately 20 hours, and for each bird, several stereotypical songs were selected. These songs were placed in succession to build six long song bouts of 30 seconds each.

#### Experimental stimuli

All birds were exposed to six different acoustic stimuli in six random ordered experiments. A collection of natural and temporally manipulated birdsongs and synthetic sounds was used. The experimental stimulus ensemble included the BOS, reversed BOS, sound that was composed of randomly ordered syllables of the bird's repertoire (random BOS), familiar conspecific song (CON), synthetic sound with power and spectro-temporal modulation spectra matched to each individual BOS but with random phase modulations (BOS ripples), and white noise (WN). The summed silence intervals between the songs within the long song bouts (30 seconds) were on average 1.2 seconds. The length of these inter-song intervals is similar to the rest intervals found in the natural bouts of undirected song that was recorded. In the following paragraph we will discuss the differences between stimuli regarding spectral modulations (i.e. the Hz frequency combinations at one point in time) and temporal modulations (i.e. the variations in amplitude over time).

**Figure 1:**
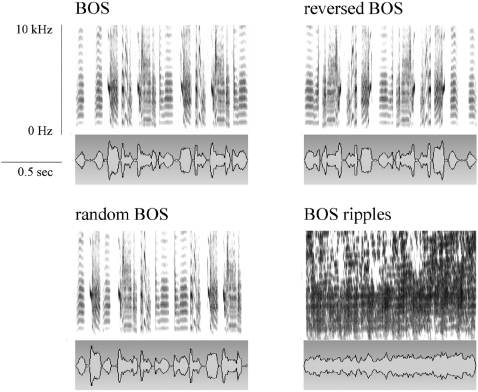
Experimental auditory stimuli. Spectrograms (top row) and oscillograms (bottom row) of an example of BOS and three temporal manipulated versions including reversed BOS, random BOS and BOS ripples. The spectrograms show that manipulations are restricted to each song separately. To obtain a better visualisation of the spectrograms, the maximum frequency shown is limited to 10 kHz (actual maximum frequency is 22 kHz).

Reversed BOS was created with Praat software (www.praat.org, Boersma P. and Weenink D.). The BOS -and thus the natural sequence of syllables- is reversed, meaning that the temporal envelope modulations are inverted but the relationship across frequency bands is preserved. In random BOS, the inter-song intervals are the same as in BOS, and the syllables are randomized within each song exemplar. Randomisation of syllables perturbs the natural sequence of syllables, but preserves the natural order of the joint spectro-temporal modulations within a single syllable. The modulation spectrum and phase of the sound envelopes on the time scale of the syllable are thus the same as found in BOS. Preservation of phase means that the relationship of the envelope of the sound in different frequency bands is preserved. BOS ripples are synthetic songs that match the spectro-temporal modulation power spectra of BOS but lack its natural modulation phase. In BOS ripples the sequence of syllables is eliminated since the relationship of the temporal modulations across frequency bands is randomized. The overall power spectrum and modulation spectrum are preserved. The BOS ripples stimulus has a total length of 30 seconds and the inter-song interval is the same as in BOS. Random BOS and BOS ripples stimuli were generated in Matlab (MathWorks, MA) with custom build routines. These routines are available upon request. More details on the synthesis of song ripples can be found in Singh & Theunissen [34] and Hsu et al. [31]. Figure 1 shows the spectrograms, and oscillograms of one example BOS and the corresponding reversed BOS, random BOS and BOS ripples. In addition, familiar CON and WN were used as control stimuli. WN has a flat power spectrum between 0 and 11 kHz, the frequency range that comprises 99% of the energy (RMS power, root mean square) calculated in our zebra finch songs. Since the noise stimulus is continuous, and BOS ripples have less complete silence in comparison with birdsong stimuli, the power of all auditory signals presented to the birds was normalized to have equal overall power (RMS). Song ripples are synthetic sounds with the same power and modulation spectrum as the natural song, but with a random phase which means that both the temporal and spectral modulations will start at random places. The effect is that the temporal profile is completely different and random, and since the onset of temporal modulations will not line up, there will be much less complete silence.

We assume that the use of sound stimuli composed of the bird's own repertoire reduces the inter-individual response differences that result from individual song preferences or song history. Stimulation with BOS is – compared to conspecific song or tutor song – less dependent on inter-individual behavioral differences, assuming that the differences in time that birds spend singing during their life don't have an effect. We assume that the biological relevance of the BOS is the same for all tested birds, making the investigation of sound processing in Field L sub-regions, NCM and CMM more accurate. Comparisons between brain responses to presentation of the natural and manipulated versions of BOS (including BOS ripples) were made to determine if the sequence of spectro-temporal modulations and phase of the sound envelopes are relevant for auditory responses in Field L sub-regions L2a, L2b and L3, and secondary regions NCM and CMM. Comparisons between brain responses to presentation of the natural BOS and familiar CON were made to determine if there is a preferential response in Field L sub-regions, NCM or CMM to either of these songs with a different biological relevance, namely song learning and maintenance (BOS) and song recognition (familiar CON).

#### Stimulation protocol

**Figure 2:**
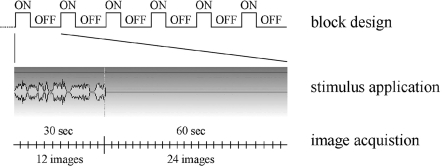
Data acquisition. Schematical representation of the auditory stimulation design. The entire paradigm was repeated 6 times with alternate presentation of the six different stimuli BOS, reversed BOS, random BOS, familiar CON, BOS ripples, and WN.

Auditory signals were presented to the birds with magnetless dynamic speakers as described in Boumans et al. [19]. Stimulus application was controlled by Presentation software (version 0.76; Neurobehavioral Systems, Albany, CA). Images were collected with a block-design paradigm consisting of 6 cycles of 12 images collected during stimulation (30 seconds) and 24 images collected during rest (60 seconds), resulting in 216 functional images (Figure 2). Each experiment, which was preceded by the acquisition of 12 dummy images to allow the signal to reach a steady state, thus took approximately 9.5 minutes. Six consecutive experiments were performed in random order during which the birds were exposed multiple times to the six different stimuli BOS, reversed BOS, random BOS, CON, BOS ripples, and WN. The average song power (average over an entire song) was set at 70 dB SPL (sound pressure level). The magnet noise was measured to be around 63 dB SPL. These sound levels were measured inside the magnet with an electret microphone.

### fMRI experiments

#### Imaging settings

MR-imaging was performed at 300 MHz on a 7 Tesla horizontal bore NMR microscope with an actively shielded gradient-insert (Magnex Scientific Ltd, Oxfordshire, UK) having an inner diameter of 100 mm and a maximum gradient strength of 400 mT/m. A Helmholtz (45 mm) and a circular RF surface antenna (15 mm) served for transmitting and receiving the RF pulses, respectively.

A set of 1 parasagittal, 1 horizontal and 1 coronal gradient-echo (GE) scout image and a set of 12 horizontal GE images were first acquired to determine the position of the brain in the magnet. Functional imaging was performed using a T_2_*-weighted single-slice GE fast low-angle shot (FLASH) sequence [field of view (FOV) = 25 mm, echo time (TE) = 14 ms, repetition time (TR) = 40 ms, flip angle = 11°, gradient ramp time = 1000 µs, acquisition matrix = 128×64, reconstruction matrix = 128×128, slice thickness = 0.5 mm]. Long gradient ramp times (1000 µs in stead of 100 µs) reduced the gradient noise to 63 dB. The functional images were acquired on a parasagittal slice in the right hemisphere from 0.25 to 0.75 mm lateral that goes through the medial extent of Field L, NCM and CM (CMM). The total acquisition time per image was 2.56 sec and the spatial resolution 195×195 µm^2^. Anatomical high resolution imaging was performed at the same position as the functional imaging slice with a T_2_-weighted spin-echo (SE) sequence (FOV = 25 mm, TE = 45 ms, TR = 2000 ms, acquisition matrix = 256×128, reconstruction matrix = 256×256, slice thickness = 0.5 mm, and eight averages).

#### Image processing

The fMRI data series were first pre-processed with MEDx software (version 3.41; Sensor Systems Inc, Sterling, KS). The following algorithms were included: 1) motion detection between subsequent images by means of a center of intensity algorithm in three directions, 2) spatial smoothing with a 3×3 pixel Gaussian convolution filter, 3) intensity normalisation with a resulting mean image intensity value of 1000.

**Figure 3:**
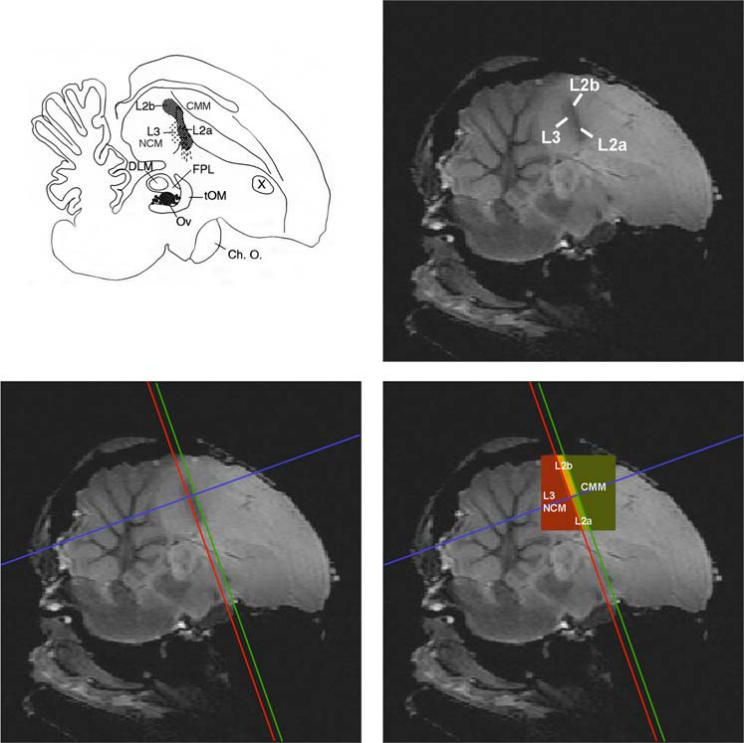
Visualisation of Field L2 on high resolution T_2_-weighted SE images and regional analysis (see online edition for color figure). The figure displays how the subfields L2a and L2b in the study of Vates et al. [46] compare to the core of the darker ellipsoid region of our anatomical high resolution MR images that corresponds to the dense fibre track that defines sub-region L2. (Schematic illustration adapted from Vates et al. [46]; anatomical MR image from Poirier et al. [50]). By drawing lines rostral and caudal from L2, and a third perpendicular line, regional analysis could be performed in a caudal/ventral region that comprises L3 and NCM, a rostral/dorsal region that comprises CMM, a dorsal region that comprises L2b and a ventral region that comprises L2a. ABBREVIATIONS, Ch. O. = Optic Chiasm; CMM = caudal medial mesopallium; DLM = medial nucleus of the dorsolateral thalamus; FPL = lateral forebrain bundle; L2a, L2b, L3 = sub-regions of Field L; NCM = caudomedial nidopallium; Ov = nucleus ovoidalis; tOM = tractus occipitomesencephalicus; X = area X.

All further image processing was performed in Matlab with custom written software. Individual analyses were performed. The pre-processed time series for each pixel were first thresholded at a level determined by a histogram of signal strengths in order to separate signal in brain regions from the signal in non-brain regions. The time series for each pixel consisted of the 12 time points acquired during stimulation and averaged over all stimulation periods followed by the 24 time points acquired during rest and averaged over all rest periods in the block design (Figure 2). A difference time signal was calculated by subtracting the first half of the rest time curve from the stimulation time curve point by point. Then 12 average signal differences were estimated by summing these difference curves for one time point, two time points, and so forth until the entire difference signal was summed. Twelve statistical tests of significance at each pixel (one sample *t*-test) were then performed for each of these twelve average signal differences. The number of time points in the sum – between 1 and 12 – that gave the highest significance over all pixels was then used to calculate the signal strength for each pixel. This number was different for the five different birds (9, 11, 11, 11, 12). The rationale for this procedure is that the observed BOLD signal was characterized by both an increase during stimulation and a decrease during rest. Moreover, both the increase and decrease started (and often peaked) at the very first time point but then, after the first or second time point, decreased monotonically to baseline, often before the end of the twelve images. Our simple procedure was designed in order to maximally detect this characteristic signal without adding the noise found at the end of the time trace. The reported signal strength for a significant pixel is then the best average signal difference divided by the global average signal difference that was obtained by averaging over all 12 time points in the sum. All non-significant pixels and all isolated statistically significant pixels were deleted from the analysis. Furthermore, we performed our analysis on a region of interest defined by the large contiguous region of activity centred around the primary auditory region. Figure 3 shows how we performed the analysis on distinct sub-regions. The darker band in the structural MR-image corresponds to the dense fibre track defining sub-region L2. By drawing lines at the rostral and caudal border of this band, and a third line perpendicular to these two lines near the center of the darker band, the activity was divided in a caudal region that comprises L3 and NCM, a rostral region that comprises CMM, and a ventral and dorsal region within Field L2 that comprises L2a and L2b, respectively.

## Results

### Localisation of BOLD responses

We chose to visualize one parasagittal slice in order to sample with a high temporal resolution the auditory regions of interest in the pallium. Our slice went through the primary auditory region Field L (in particular sub-areas L2a, L2b and L3) and secondary auditory (or associative) regions NCM and CMM. This slice was orthogonal to our previous one used in the characterisation of BOLD responses in the avian auditory forebrain [29], allowing us to investigate responses in caudal L2b and CMM that were not examined in the previous study.

**Figure 4:**
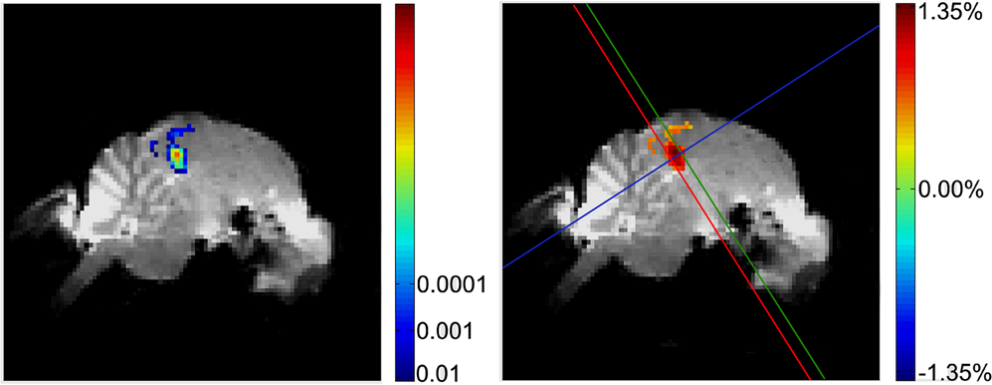
Average BOLD signal of one example bird (see online edition for color figure). The images illustrate the typical activation pattern that was found in all experimental birds. The signal shown here is for all sounds presented and all brain images averaged together. The left panel shows the *P*-values of significant activated pixels, the right panel shows the signal strength relative to the mean signal difference. The three lines show the division in regions of interest conform Figure 3.

For all sound stimuli, we found strong activation of the primary auditory region, Field L. Figure 4 shows a typical example of activation, for all sounds, averaged. The peak of the BOLD activity was mostly in precise register with the core of the darker band in the structural MR-image that corresponds to the dense fibre track defining sub-region L2 as shown in Figure 3. Given the larger spread of activity in the caudo-rostral dimension, we conclude that the BOLD activation that we measured also extends to the neighbouring regions L3 and NCM on the caudal/ventral side, and CMM on the rostral/dorsal side.

### Auditory stimulus selectivity

Observation of the activation and its regional variability for all experimental sounds separately showed that specific sounds were not able to activate regions other than those significantly activated after averaging across all stimuli. For this reason, we performed all of our analyses on the ensemble of voxels that were significantly activated for all sounds averaged together.

**Figure 5:**
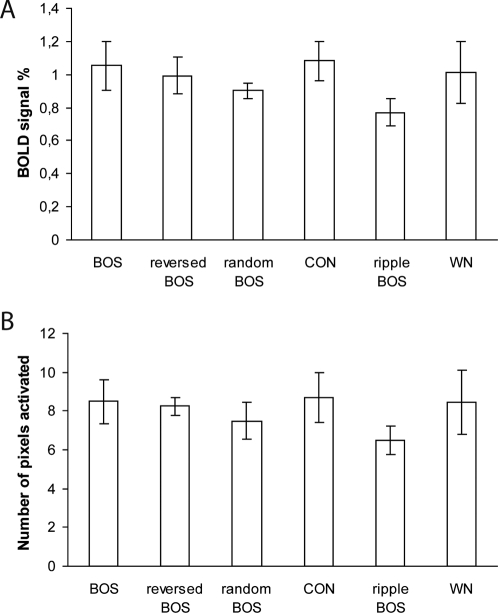
Stimulus and regional selectivity. (A) The average BOLD signal and (B) the average number of pixels activated in the four regions of interest L2a, L2b, L3/NCM and CMM together with exposure to BOS, reversed BOS, random BOS, CON, BOS ripples and WN. All means are represented with their corresponding standard errors (SEM).

If the auditory system is tuned to the natural sequence of spectral and temporal modulations found in CON, and in particular BOS, we would expect to find a decrease in activity to the manipulated song stimuli that differ in spectro-temporal modulations sequence and/or phase. Figure 5A shows the average BOLD signal (i.e. the average percent signal intensity change between stimulation and rest periods) with exposure to BOS, reversed BOS, random BOS, CON, BOS ripples and WN. To determine whether differential responses exist between stimulus types, we performed a statistical analysis on the voxels that were significantly activated for all sounds averaged together. An ANOVA for repeated measures with the average BOLD response amplitude as dependent variable and with Stimulus as repeated factor showed that there was no significant effect of Stimulus (F_5_ = 1.022; P = 0.431). Figure 5B shows the average number of pixels activated with exposure to BOS, reversed BOS, random BOS, CON, BOS ripples and WN. An ANOVA for repeated measures with the average number of pixels activated as dependent variable and with the Stimulus as repeated factor showed that there was no significant effect of Stimulus (F_5_ = 1.269; P = 0.316).

### Variation in response between auditory regions

Because the analysis performed on the whole activated cluster could mask different functional activations between different auditory (sub-) regions, we chose to perform a regional analysis by dividing the auditory activity into four parts containing L2a, L2b and the regions caudal/ventral and rostral/dorsal to L2. The caudal/ventral region includes L3 and NCM respectively, while the rostral/dorsal region includes CMM.

The four regions of interest include different amounts of significant activated pixels. This observation was quantified by counting the number of significant pixels in the subdivisions L2a, L2b, L3/NCM and CMM. The significant pixels were mostly found in the ventral auditory regions covering L2a and part of L3/NCM. The average number of significantly activated pixels was the greatest for L2a (21 pixels or 0.8 mm^2^), followed by L3/NCM (16.2 pixels or 0.62 mm^2^), with the least activated pixels for CMM (7.8 pixels or 0.3 mm^2^) and L2b (7.2 pixels or 0.27 mm^2^). A one-way ANOVA with the count of significant activated pixels as dependent variable revealed a significant effect of the factor Region (F_3_ = 33.456; P<0.001). Post-hoc tests corrected for multiple comparisons (Bonferroni) showed differential amounts (all P<0.01) between any two regions, with the exception of the pair L2b and CM.

**Figure 6:**
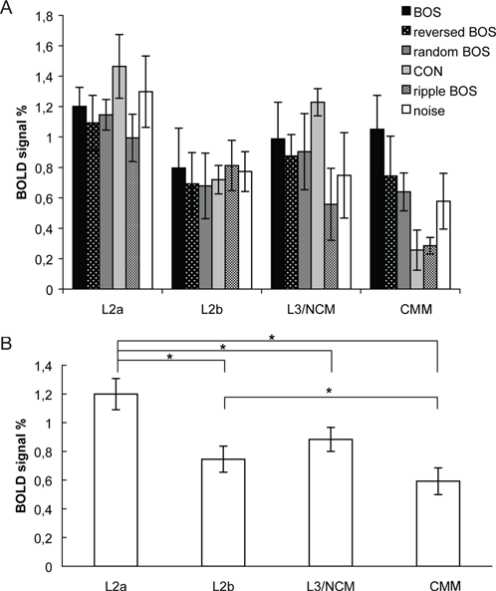
Regional selectivity. This figure shows the average BOLD signal in the four regions of interest L2a, L2b, L3/NCM and CMM, for all sounds separately (A) and averaged (B). All means are represented with their corresponding standard errors (SEM). Asterisks (*) indicate significant differences.

An ANOVA for repeated measures with the average BOLD response amplitude as dependent variable and with Region and Stimulus as repeated factors revealed a significant effect of Region (F_3_ = 9.071; P = 0.002). No significant effect of Stimulus (F_5_ = 0.951; P = 0.470), and no significant interaction between Region and Stimulus (F_15_ = 1.567; P = 0.111) was observed. An ANOVA for repeated measures with the average BOLD response amplitude averaged over the different stimulus types as dependent variable and with only Region as repeated factor, showed again a significant effect Region (F_3_ = 9.071; P = 0.002). Post-hoc tests corrected for multiple comparisons (Bonferroni) between the four regions showed differential signal strengths between region L2a and the regions L2b (P = 0.034), L3/NCM (P = 0.028), and CMM (P = 0.025), and between region L2b and region CMM (P = 0.034). There was a non-significant trend between regions L3/NCM and CMM (P = 0.072). Figure 6 shows the average BOLD signal in the four regions of interest L2a, L2b, L3/NCM and CMM, for the different stimuli separately (Figure 6A) and averaged (Figure 6B).

## Discussion

### Nature of the fMRI signal and comparison with conventional techniques

BOLD fMRI allows to measure changes of the oxy-hemoglobin (HbO2) / deoxy-hemoglobin (Hb) ratio in brain tissue. During neural activation, after a short decrease of oxygen level due to oxygen consumption by neurons, neuro-vascular mechanisms over-compensate this consumption by increasing the cerebral blood flow and the cerebral blood volume, resulting in a net increase of the HbO_2_/Hb ratio [35]. The BOLD signal is thus a correlate of the global activity of large pools of neurons. BOLD fMRI signal is best correlated with local field potentials [36], reflecting the synaptic and post-synaptic activity of neurons, and does not allow a distinction between excitatory and inhibitory neurons. To be detectable with fMRI, selectivity has to be expressed by a substantial number of neurons, concentrated at one location and presenting the same properties. As a consequence, results obtained by this technique can be quite different from results based on neuronal firing rates or on immediate early gene expression and do not allow to make inference about selectivity at the level of individual neurons. The fMRI technique rather brings complementary information about common properties of large pools of neurons and has the advantage of allowing to test several stimuli in the same bird in different regions at the same time.

### Selectivity for the bird's own song

Selectivity for the bird's own song was investigated by comparing BOS versus CON stimuli. When performed on the big cluster of voxels activated by all stimuli together, this comparison revealed no statistical differences between the two stimuli. The activity triggered by BOS stimuli was even slightly weaker that the one induced by CON stimuli, indicating that the absence of BOS selectivity cannot be due to a lack of statistical power. BOLD fMRI has been shown to be a relevant tool to detect differences between conspecific songs and artificial stimuli [19] and manipulated versions of conspecific songs [29]. In a subsequent study (C. Poirier unpublished observation), fMRI was also found to be able to detect BOS selectivity in the song control system, including HVC. The absence of significant BOS selectivity observed in the present study is thus not due to limitations inherent to the fMRI technique. This result rather indicates that the auditory-responsive region as investigated here, shows no selectivity for BOS at the global neural level. With a similar analysis, Voss et al. [33] also failed to find a statistically significant difference between BOS and CON responses in their fMRI study of mildly sedated zebra finches.

Because this absence of BOS selectivity in the whole cluster did not preclude selectivity in auditory sub-regions, we also compared the activation elicited by BOS and CON in each sub-region. This second analysis confirmed the absence of significant BOS selectivity in the different sub-regions but nevertheless revealed a non-significant trend for BOS selectivity in CMM. The absence of significant difference in L2a and L2b confirms the lack of BOS selectivity of L2a observed in our previous experiment [29]. Electrophysiological experiments previously looked for BOS selectivity in Field L neurons of the zebra finch [25], [37], [38]. These experiments reported a lack of selectivity in the majority of neurons. All together, these results suggest that the primary auditory cortex does not present BOS selective properties neither at the neuronal level nor at a more global neural level.

Tutor song-induced neuronal activation in the NCM is related to the strength of song learning [9], [10], [14], [17]. It suggests that this auditory region may contain the neural substrate for a representation of tutor song memory. Since BOS can be very similar to the tutor song, one might also expect that NCM would show a preference for the BOS. However, Terpstra et al. [14] did not find such learning-related neuronal activation in the NCM when zebra finch males were exposed to BOS. Consistent with this, the present results did not show stimulus-specific BOLD response differences to BOS versus CON. However, the absence of significant BOS selectivity in the region of interest L3/NCM should be interpreted carefully since it mixes two regions L3 and NCM that could differ functionally. A non-significant trend for BOS selectivity was found in CMM. The auditory regions were found to be hierarchically organised in terms of signal strength, with CMM more weakly activated than L2a and L2b. This weaker activity induces a reduced probability to detect any selectivity in this region. Previous electrophysiological recordings have shown that CLM neurons, including those that are functionally connected to HVC, exhibit a lack of BOS selectivity [37], [38]. However, a recent study has found that few excitatory neurons of CMM were BOS selective [39]. In songbirds, determining which regions show BOS selectivity is important, as neurons sensitive to self-generated vocalisations could mediate auditory feedback that is necessary for song learning and maintenance, similarly to speech in humans. In humans, secondary or tertiary auditory regions are suspected to be involved in this auditory feedback control of speech [40], review: [41]. The BOS selectivity found in songbird CMM [39] is congruent with the involvement of secondary and tertiary human auditory regions in auditory feedback. To be conclusive, these issues about BOS selectivity in songbird NCM and CMM will require additional experiments.

### Selectivity for temporal characteristics of the bird's own song

The comparison of the neural activity elicited by BOS and manipulated BOS (reversed, random BOS) revealed no significant difference in the auditory-responsive cluster. However, since the activation triggered by BOS stimuli is slightly higher, we cannot rule out the hypothesis that the lack of significance reported here is due to a weak statistical power. The same analysis performed in each sub-region confirmed the absence of a significant difference despite a non-significant trend observed at least in CMM.

Electrophysiological recordings from neurons in HVC and Field L revealed that neurons in Field L showed much less sensitivity to manipulations of the auditory temporal context than neurons in HVC [25]: HVC neurons responded strongly to the forward song but weakly to the reversed song and to the song with the syllables or sub-syllables in reverse order while neurons in L1, L2a, L2b and L3 responded strongly to a forward song, a reversed song, and to the syllables and sub-syllables in reverse order. In another study [37] investigating the same selectivity in sub-regions of Field L (L, L1, L2a, L2b and L3) and CLM, a weak preference for BOS over temporally manipulated BOS was found. Despite a lack of significant differences between regions, the results suggest that this selectivity is slightly higher in CM and almost absent in L2a. All together, electrophysiological and fMRI results point to a lack or a very weak sensitivity for the temporal order of the BOS in Field L while CM may present an intermediary selectivity (as compared to HVC).

### Selectivity for conspecific songs

Conspecific selectivity was investigated by comparing conspecific stimuli (including CON and BOS) with two artificial stimuli, ripple BOS and white noise. Song ripples were synthetic sounds that matched the spectro-temporal modulation power spectra of BOS but with a random modulation phase. This comparison revealed no significant differences in the auditory-responsive cluster with a non-significant trend of selectivity for conspecific stimuli over ripple BOS. A weak statistical power is thus a possible explanation for the lack of a significant difference. The same comparison performed on each sub-region confirmed this absence of a significant difference. The non-significant trend for BOS versus ripple BOS was found to be the most pronounced in CMM.

Neuronal selectivity for conspecific vocalisations has been found in numerous animal models as non-human primates, cats, mice, bats, and frogs. This selectivity is generally found in the secondary auditory cortex [42], [43]. In birds, electrophysiological recordings have also shown some selectivity for conspecific song over matched synthetic sounds including ripples and white noise in L1, L2a, L2b, L3 and CLM [26] in excitatory but not in inhibitory neurons. Since fMRI signal reflects the global activity of large pools of inhibitory and excitatory neurons, this lack of significant differences between conspecific songs and artificial sounds could be due to this heterogeneity of neuronal selectivity between excitatory and inhibitory neurons. Artificial stimuli and especially white noise contain more numerous frequencies and could thus activate a large number of auditory neurons. The neuronal selectivity for conspecific song compared to noise was also found to increase when the onset response to noise was removed [26]. Since fMRI signal integrates neural activity over several seconds, it does not allow to distinguish between onset and sustained responses. This strong onset response to noise may thus have limited putative differential response between conspecific songs and noise by participating to a high neural activity elicited by noise in the present experiment. However, due to the short length of onset responses as compared to sustained responses, fMRI signal mainly reflects sustained responses [44] and the contribution of the onset response is limited.

### Region-specific differences in the auditory telencephalon

Despite a lack of clear evidence for selectivity in the auditory cortex, our results demonstrate a clear hierarchical organisation from L2a to L2b and from L2b to CMM in term of signal strength. Auditory information in the avian brain travels from the cochlear nuclei through the midbrain to the thalamic nucleus Ovoidalis (Ov) and from there to the telencephalic Field L. The main Ovoidalis thalamo-recipient zone is L2: L2a receives input from Ov “core” and from other sub-regions of Field L whereas L2b receives inputs from a ventro-medial sub-division of the core and from L2a. The sub-regions L1 and L3 are immediately adjacent to L2a, and receive L2a input as well as a smaller amount of thalamic input from the Ov “shell” region. The presence of subfields is based on differences in cytoarchitecture and connectivity [45], [46]. Field L projects to the secondary auditory areas NCM and CM in the telencephalon, from subfields L2a and L3, and from subfields L1, L2b and L3 respectively. NCM also receives input from CMM and from Ov [46].

The decrease of fMRI signal intensity observed from L2a to CMM trough L2b could be due to two distinct phenomena: it can indicate a decrease of the neuronal activity of each individual neuron or a decrease of responding neurons resulting from an increasing heterogeneity and specialisation of individual neurons. Without excluding the first hypothesis, electrophysiological recordings rather support the second one. A decrease of response strength was indeed observed from L2a to CLM through L2b in multi-unit recordings but became less obvious in single-unit recordings [30], [38].

One interesting result, which is explicitly described in this analysis, is the difference in signal strengths between the two subareas of L2: L2a is significantly more activated than L2b. A previous electrophysiology study that used pure tones already indicates that Field L is not a functionally homogeneous region [47]. This characteristic seems to be shared with non-human primates since different sub-regions of the primary auditory cortex of macaques present different functional characteristics in term of tonotopy, latency and frequency tuning [48], [49]. By highlighting functional differences using natural stimuli, the results of the present experiment suggest that these differences may play an important role in the encoding and processing of behaviourally relevant auditory signals. The stronger BOLD response in L2a correlates with its central place in the hierarchy of ascending processing stages found in the auditory telencephalon [46]. L2b receives input from a smaller medial region of Ov core as well as from L2a. L2b could therefore already play a distinct functional role than L2a.

### Conclusion

Our results show a clear hierarchical organisation in terms of signal strength between L2a, L2b and CMM but no obvious selectivity for BOS or for temporal characteristics of BOS in these regions. Especially, we found that the primary auditory sub-regions L2a and L2b do not show any preferential responses (at the global neuronal population level) to BOS or CON. Selectivity in the secondary auditory regions is less clear and will require additional investigation. It should also be noted that the present fMRI experiment does not exclude the possibility of BOS selectivity emergence in other areas of the ascending auditory pathway, e.g. in lateral parts of Field L or in the auditory nuclei of the midbrain and of the thalamus. These issues should be the object of future investigations.
